# Synthesis, Radiolabeling and Biological Evaluation of ^99m^Tc-labeled Deoxyglucose Derivatives for Molecular Imaging 

**Published:** 2013

**Authors:** Masoud Sadeghzadeh, Ghorbanali Charkhlooiea, Fariba Johari Daha

**Affiliations:** a *Radioisotope Research Group, NSTRI, P.O.Box:11365-3486, Tehran, Iran.*; b *Young Researchers and Elite Club, Karaj Branch, Islamic Azad University, Karaj, Iran. *

**Keywords:** ^99m^Tc, Radiopharmaceuticals, Glucosamine derivatives, ^99m^Tc-tricarbonyl complex, Molecular imaging

## Abstract

Two deoxyglucose (DG) derivatives, (*α,β*)-2-deoxy-2-amino(ethylcarbamate)-D-glucose (ECB-DG) and (*α,β*)-2-deoxy-2-amino(1,2-dihydroxypropyl)-D-glucose (DHP-DG), were synthesized and radiolabeled successfully with [^99m^Tc(H_2_O)_3_(CO)_3_]^+^ complex. [^99m^Tc]-ECB-DG and [^99m^Tc]-DHP-DG complexes were prepared (96% and 93% radiochemical purities respectively) by using 46 mCi of Na^99m^TcO_4_ in 1 mL saline. Radio-HPLC analysis of [^99m^Tc]- ECB-DG at pH = 7.4, revealed that labeling with ^99m^Tc leads to formation of one radiochemical species with t_R_ = 381 second. Three radiochemical species, Na^99m^TcO_4_, [^99m^Tc]-DHP-DG and [^99m^Tc(H_2_O)_3_(CO)_3_]^+ ^complexes with t_R_ = 342 sec, t_R_ = 567 sec and t_R_ = 1586 sec respectively, were obtained when [^99m^Tc]-DHP-DG complex evaluated by HPLC. Biodistribution of two complexes were studied on normal mice at 10, 30 and 60 min post-injections. Compared to the ^18^F-FDG, [^99m^Tc]-ECB-DG displayed a 2.8-fold reduction in brain uptake (1.7 ± 0.2 versus 0.61% ± 0.09) ,whereas [^99m^Tc]-DHP-DG just showed 1.9-fold reduction in heart uptake (2.2 ± 0.05 towards 1.16±0.10) at 1 h post-injection. On the basis of our results, it seems that ECB-DG and DHP-DG analogues could be used as brain and heart imaging agent respectively.

## Introduction

Carbohydrates are of primary importance, because they are sources of energy for living organisms. Therefore, the design of carbohydrate-appended ^99m^Tc complexes for potential use in nuclear medicine is of considerable interest (1). The accurate and early non-invasive detection of malignant disease is an important factor in the treatment and prognosis of a patient with cancer. Improvements in tumor radionuclide imaging depend on the development of more tumor-specific radiopharmaceuticals (2). One compound that is very useful for the detection of tumors and metastatic tissue is 2-deoxy-2- [^18^F]fluoro-D-glucose (FDG), which is imaged by positron emission tomography (PET) (1). Because the production of Flourine requires a cyclotron, and the isotope has a short half-life (110 min) , its utility is somewhat limited. Hence, this problem led to the search for alternatives to FDG by utilizing radionuclide that decay by a process that can be imaged by single photon emission computed tomography (SPECT). SPECT is much more prevalent and enabling the use of ^99m^Tc, which has ideal nuclear properties (t_1/2 _= 6.01 h, *γ *= 140 keV). It is also the most widely used radioisotope in nuclear medicine (3). 

For these reasons, we seek a ^99m^Tc SPECT tracer that will mimic the biodistribution of FDG for molecular imaging. Our approach is to attach to glucose a chelating ligand that, in a subsequent reaction, will bind the radioisotope ^99m^Tc. A metal-chelate could be preformed and then attached to glucose. To mimic the properties of FDG, it is imperative that the effects of the tracer group on the properties of the glucose molecule be minimized. Existing ^99m^Tc labeled glucose derivatives fail this criterion: they are either ionic or have relatively high molecular weight (*i.e. *carry two glucose moieties) (4, 5). 

Stable core structures have found utility in the chemistry of Tc due to the wide range of accessible oxidation states (Tc(-1) to Tc(+7)). For example, the {M (CO**)**_3_}^+^ (M = Tc/Re) core has garnered significant interest since its development by Jaouen (6) and Alberto and co-workers (7). This organometallic core offers advantages in terms of stability, kinetic inertness, and size. The low-spin d^6^-electron configuration and the stability of CO ligands to substitution protect the metal center from further ligand substitution and/or oxidation. For the above reasons there has been widespread interest in the development of target-specific radiopharmaceuticals exploiting the {M (CO**)**_3_}^+ ^(Tc/Re) core. In neutral complexes with simple N, O donors the *fac*-[^99m^Tc (I)-(CO)_3_] core possesses intermediate lipophilicity, an advantage in living systems (8). The [^99m^Tc (CO)_3_]^+^ carbonyl moiety is extremely interesting due to its high *in-vitro *and *in-vivo *stability when it is connected to various biomolecules. It has been reviewed in detail for its use in the second generation of single photon emission computed tomography (SPECT) radiopharmaceuticals (4, 8-12). Introduction of the Tc (I) can be achieved by convenient use of the [^99m^Tc(H_2_O)_3_(CO)_3_]^+^ complex ,which can be synthesized easily from a commercially available kit formulation (Isolink1, Mallinckrodt) following Alberto’s method of synthesizing [^99m^Tc(CO)_3_(H_2_O)_3_]^+^ from [^99m^TcO_4_]^-^ in aqueous solution (13).

Glucosamine (2-amino-2-deoxy-D-glucose) is a highly attractive scaffold for a glucosyl ligand, because the amine acts both as a potential coordination site and as a useful target for further functionalization. Furthermore, there is much evidence in the literature which suggest that *N*-functionalized glucosamines show activity with GLUTs (glucose transporters) and hexokinases, the enzymes that are most closely associated with the metabolism of FDG, even when the functional group is large (2, 14). For example, Yang *et al. *reported that ethylenedicysteine-deoxyglucose (EC-DG) as an *N*-functionalized glucosamine, shows activity with hexokinases. Their findings suggested that hexokinase-catalyzed phosphorylation had occurred with EC-DG, 2-deoxy-D-glucose, 2-fluorodeoxyglucose (FDG), and glucose. Whether EC-DG and 2-deoxy-D-glucose use different glucose transporters,both of them need to be further investigated (5). In addition, it has been confirmed that there are at least two mechanisms for the cellular processes of glucosamine. The first mechanism resembles the cellular process mechanism of a glucose transporter system. In the second mechanism, glucosamine enters cells and forms glucosamine-6-phosphate directly in the additional transcriptional pathways (15-19). Lots of ^99m^Tc-labeled glucose and D-glucosamine derivatives have been synthesized in order to develop one subrogate in SPECT for [^18^F]-FDG in PET (2, 4, 5, 11, 12, 20-24).

## Experimental

All chemicals were purchased from Aldrich and Merck companies and used without further purification. Carbon monoxide was obtained in the form of refillable canisters (0.5 L) from M/s Alchemie Gases & Chemicals. ^99m^TcO_4_^-^ was eluted from an in-house ^99^Mo/^99m^Tc column generator using normal saline. HPLC analyses were performed on a JASCO 880-PU HPLC (Tokyo, Japan) equipped with a Ray test-Gabi gama ray detector. A Polygosil 5 μm RP-C18 analytical column (reverse phase) with dimensions of 250×4.6 mm was used. IR spectra were taken films KBr pellets on a Bomem spectrometer (Bgrams). ^1^H NMR and ^13^C NMR spectra were run on Bruker (DRX-500 Advance) spectrometer at 500 (^1^H NMR) and 125 (^13^C NMR) MHz, in D_2_O as solvent. Chemical shift has been expressed in ppm rel. to Me_4_Si as internal standard. Mass spectra were recorded on Instrument Mass HP (Agilent technologies) 5937 mass selective detector using electron ionization (EI) in-positive mode. All radioactivity measurements were carried out using NaI (Tl) scintillation counter.

*Synthesis of (α,β)-2-deoxy-2-amino (ethylcarbamate)-D-glucose (1)*

Ethylchloroformate (0.33 mL, 3 mmol) was added dropwise to an ice-cooled solution of (*α,β*)-2-amino-2-deoxy-D-glucose hydrochloride (0.5 g, 3.07 mmol) and sodium hydrogen carbonate (0.49 g, 5.83 mmol) in water (5 mL). The mixture was stirred in an ice bath for 2 h and then at room temperature overnight then dioxane (20-30 mL) was added to the mixture. The reaction mixture was filtered, and the solvent was removed. The product was purified by flash column chromatography on silica gel using CH_3_OH/ethyl acetate (2:8) as eluant; yield 70%; mp: 178-180 ºC; IR (KBr): ν (cm^-1^) 3500-3250 (–OH, -NH), 2948 (CH), 1686 (C=O), 1544 (–NH), 1132 (C-O); ^1^H NMR (CDCl_3_): δ ppm 1.1 (t, 3H, -CH_3_, *J *= 11.7 Hz,), 3.3-3.8 (m, 6H), 4.0 (q, 2H, -CO_2_CH_2_-, *J *= 11.7 Hz,), 4.56 (d, 1H, -OCHCH_2_OH, *J *= 13.7 Hz,), 5.1 (bs, 1H, anomeric –OCHOH); ^13^C NMR (CDCl_3_): δ ppm 13.8, 55.4, 60.5, 60.8 (C of another conformer), 69.9, 70.0 (C of another conformer), 71.1, 71.5 (C of another conformer), 74.0, 75.9, 91.3, 158.6; Mass (EI, 70 eV) m/z: 251 (M^+^), 220, 205, 144, 88, 57.

*Synthesis of (α,β)-2-deoxy-2-amino(1,2-dihydroxypropyl)-D-glucose (*2*) *

3-Chloro-1,2-propandiol (0.049 mL, 0.5 mmol) was added to a solution of (*α,β*)-2-amino-2-deoxy-D-glucose hydrochloride (1.0 g, 6.13 mmol) and powdered sodium carbonate (0.0934 g, 0.9 mmol) in water (10 mL), and then the mixture was heated at 110 ºC with stirring. After 10 h, the mixture was cooled to reach the room temperature, and ethanol (20-30 mL) was added. Then the reaction mixture was filtered, and ethanol was removed. The residue was extracted with ethyl acetate, and recrystallized in water yielded DHP-DG as a colorless crystal; yield 71%; mp: 198-199 ºC; IR (KBr): ν (cm^-1^) 3500-2500 (–OH, -NH), 1538 (–NH); ^1^H NMR (CDCl_3_): δ ppm 3.1 (d, 2H, -NHCH_2_CH(OH)-, *J *= 5.9 Hz,), 3.2 (d, 2H, -CH(OH)CH_2_OH, *J *= 5.9 Hz,), 3.4-3.8 (m, 7H), 4.83 (d, 1H, -OCHCH_2_OH, *J *= 14.0 Hz,), 5.3 (d, 1H, anomeric –OCHOH, *J *= 5.9 Hz,); ^13^C NMR (CDCl_3_): δ ppm 54.3, 56.7, 60.4, 60.5 (C of another conformer), 69.6, 69.7 (C of another conformer), 71.5, 71.9 (C of another conformer), 76.1, 89.1, 92.7, 94.1; Mass (EI, 70 eV) (*m/z*): 253 (M^+^), 72, 59, 36. 

*Preparation of* ^99m^ *Tc tricarbonyl precursor (3)*

Synthesis of [^99m^Tc (H_2_O)_3_(CO)_3_]^+^ complex was carried out according to the previously reported method (10). Complex 3 was prepared using a modification of the procedure described by Alberto et al. A 10 mL vial containing Na_2_CO_3_ (5 mg), NaBH_4_ (4 mg) and sodium potassium tartarate (10 mg) was capped with a rubber stopper and then flushed with a stream of CO gas (99.5%) at room temperature for 10 min. One milliliter of sodium pertechnetate (Na^99m^TcO_4_) with up to 20-100 mCi were added by a syringe and then heated to 75 °C for 30 min. After rapid cooling down to room temperature, 0.3 mL of 0.1 M HCl was added to decrease the pH (pH = 9.5-10). The radiochemical purity was > 98% which was determined by HPLC. 

*Preparation of [* ^99m^ *Tc]-ECB-DG (4) and [* ^99m^ *Tc]-DHP-DG (*5*) complexes *

Labeling was achieved by mixing an aliquot (0.3 mL) of the [^99m^Tc(H_2_O)_3_(CO)_3_]^+ ^precursor with ECB-DG or DHP-DG (38 mg) in PBS (pH = 7.4, 1 mL) and incubating at 75 ºC for 30 min. [^99m^Tc]-ECB-DG or [^99m^Tc]-DHP-DG complexes were carried out by HPLC using C18 reverse phase column. HPLC solvents consisted of 0.1% trifluoroacetic acid in water (solvent A) and acetonitrile (solvent B). Samples were analyzed with linear gradient method (100% solvent A to 100% solvent B over 30 min.). The test solution (20 μL) was injected into the column and the elution was monitored by observing the radioactivity profile. The flow rate was maintained at 1 (mL/min). 

*Stability studies*

The radioactive [^99m^Tc]-ECB-DG and [^99m^Tc]-DHP-DG complexes were tested *in-vitro *in 1N physiological phosphate buffer (PBS, pH=7.4) and human serum at 37 °C for 3, 7, 10 and 24 h. The stability was assayed by monitoring the HPLC elution profile and determined the radiochemical purity after incubation at 37 °C until 24 h. To determine *in-vitro *serum stability, 150 μL of radiolabeled complexes were incubated with 1 mL human serum at 37 ºC for mentioned times. A 1 mL ethanol was added to the aforementioned solutions. The precipitates were separated by centrifugation and the supernatants were injected in HPLC to determine the stability of these complexes. 

## Results and Discussions

**Figure 1:**
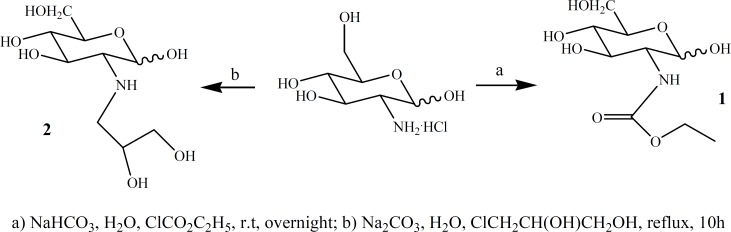
Synthesis of ECB-DG (1) and DHP-DG (2).

In the context of our general interest for the synthesis of glucosamine derivatives for molecular imaging (17), we herein reported two new 2-deoxy-D-glucose (DG) derivatives, *i.e*: (*α,β*)-2-deoxy-2-amino (ethylcarbamate)-D-glucose (ECB-DG) and (*α,β*)-2-deoxy-2-amino(1,2-dihydroxypropyl)-D-glucose (DHP-DG), which synthesized in good yields (Figure 1) and then labeled with high radiochemical purity by [^99m^Tc(H_2_O)_3_(CO)_3_]^+^ successfully. Radio-HPLC analysis of [^99m^Tc]-DHP-DG at pH = 7.4, revealed that labeling with ^99m^Tc resulted in the formation of three radiochemical species (Na^99m^TcO_4_ with t_R_ = 342 sec, [^99m^Tc(CO)_3_(H_2_O)_3_]^+ ^complex with t_R_ = 1586 sec and [^99m^Tc]-DHP-DG [radiochemical purity = 93%] with t_R_ = 567 sec with different HPLC-profiles. On the other hand, [^99m^Tc]-ECB-DG complex [pH = 7.4, radiochemical purity = 96%] displayed radio-HPLC chromatogram with t_R_ = 381 sec. This report describes the synthesis, radiolabeling with ^99m^Tc and preliminary biodistribution study on normal mice. 

*Chemistry*

The chemistry necessary for the synthesis of the ECB-DG and DHP-DG were mainly based on organic chemistry techniques. The functionalization of glucosamine at C-2 position with ethylchloroformate and 1, 2-propandiol moieties were accomplished in one step (Figure 1). [^99m^Tc (H_2_O)_3_(CO)_3_]^+^ has proved to be an excellent agent for labeling different kind of ligands. It can be formed in high yield directly from generator eluted pertechnetate in aqueous solution. As three coordinated water are labile, they could be exchanged readily with a variety of mono-, bi- and tridentate ligands forming complexes. The major advantage of using the carbonyl precursor is that high specific activity labeling of biomolecules can be obtained. 

*Radiolabeling and HPLC characterization*

**Figure 2:**
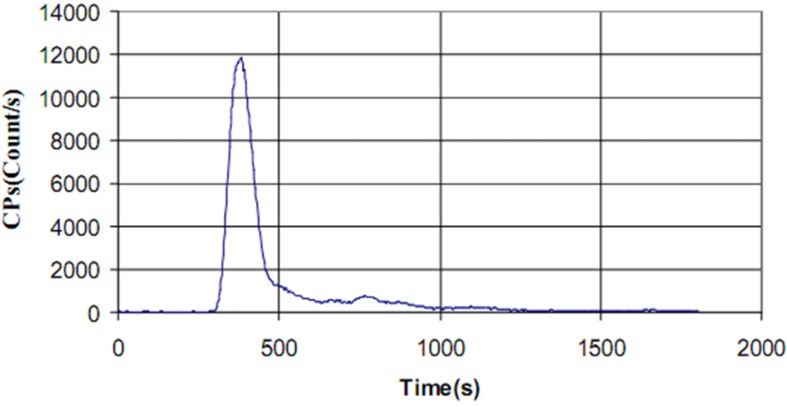
Radio-HPLC chromatogram of the [^99m^Tc]-ECB-DG with t_R_= 381 sec

**Figure 3:**
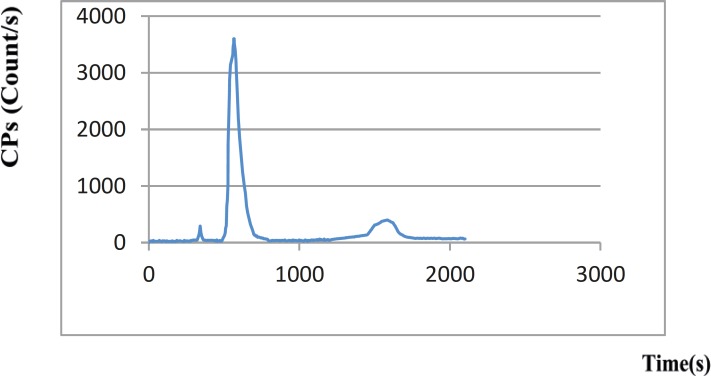
Radio-HPLC chromatogram of the [^99m^Tc]-DHP-DG for Na^99m^TcO_4_ with t_R_ = 342 sec, [^99m^Tc]-DHP-DG complex with t_R_ = 567 sec and [^99m^Tc (H_2_O)_3_(CO)_3_]^+^ complex with t_R_ = 1586 sec

[^99m^Tc]-ECB-DG and [^99m^Tc]-DHP-DG complexes showed different HPLC chromatograms in the neutral pH conditions tested. After 30 min of incubation of the [^99m^Tc]-ECB-DG complex at 75 ºC, cooling and then injection of the sample to column, we found only one peak with a retention time of 381 sec for [^99m^Tc]-ECB-DG and a small amount of impurities with a retention time of 750-800 sec (Figure 2). However, injection of the [^99m^Tc]-DHP-DG complex resulted in different HPLC chromatogram profile. Three radiochemical species, Na^99m^TcO_4_, [^99m^Tc]- DHP-DG and [^99m^Tc(H_2_O)_3_(CO)_3_]^+^ complexes with retention time 342 sec, 567 sec and 1586 sec respectively, were obtained when [^99m^Tc]- DHP-DG complex evaluated by HPLC (Figure 3). The comparable retention times of [^99m^Tc]- ECB-DG and [^99m^Tc]-DHP-DG complexes indicate that the lipophilicity has not been drastically changed by the change of organic chelator in the glucosamine structure. The [^99m^Tc]-ECB-DG showed a slightly shorter retention time on RP-HPLC and thus relatively higher polarity. 

*Labeling with* ^99m^ *Tc*

^99m^Tc (I) tricarbonyl precursor, complex 3, was successfully prepared with 98% radiolabeling using a modification of the procedure described by Alberto *et al. *The complex was stable (> 62%) for approximately 3 h. After this time, decomposition of the complex to Na^99m^TcO_4_, [^99m^Tc(H_2_O)_3_(CO)_3_] and other carbonyl species was observed. The ^99m^Tc complex of the ECB-DG and DHP-DG were obtained by addition of 3 at biological pH value (pH = 7.4). On the basis of optimization studies on parameters for labeling, it was observed that the radiochemical purities of these complexes were dependent on the pH of reactions*.*

Maximum yields of labeling (~93%) could be achieved when the reactions were carried out at biological pH, but cleavage of the glucosamine moiety was observed when the labeling was carried out in alkaline condition. Although, we reached to better resolutions, but the expanding of the peaks may be took place because of the presence of *α,β*-anomeric carbons in compounds 1 and 2. However, these complexes were stabled for 7 hours in 1 N of PBS as well as human serum at 37 ºC (Table 1).

**Table 1:** Stability studies of [^99m^Tc]-ECB-DG and [^99m^Tc]-DHP-DG complexes remaining after incubation at 37 °C in 1N physiological phosphate buffer (PBS, pH=7.4) and human serum for 3, 7, 10 and 24 h determined using HPLC

**Time (h)**	**Incubation in 1N PBS**				**Incubation in human serum**			
	3	7	10	24	3	7	10	24
% of [^99m^Tc]-ECB-DG remaining	>98	52	31	17	95.5	63	28	20
% of [^99m^Tc]-DHP-DG remaining	94	46.5	26	15	90	50	25	18

*Biodistribution in mice*

The animals were housed at The Animal Center of Nuclear Science & Technology Research Institute in Iran. Biodistribution experiments of injected radiolabeled sugar were performed on normal mice (25–35 g). Each animal was cited in specific container and then was anesthetized with ketamine/xylazine and injected intravenously with [^99m^Tc]-ECB-DG and [^99m^Tc]-DHP-DG complexes (0.3 mCi) in 0.1-0.3 mL of saline into a lateral tail vein. At 10, 30, and 60 min post-injection, blood samples were drawn by cardiac puncture, and the mice were sacrificed thereafter by cardiectomy while under ketamine/xylazine anesthesia. The organs of interest were then excised, blotted with tissue paper, weighed, and the radioactivity was counted. The percent of injected dose/organ was determined by comparison of the tissue radioactivity with suitably diluted, known quantity aliquots of the injected dose.

**Figure 4:**
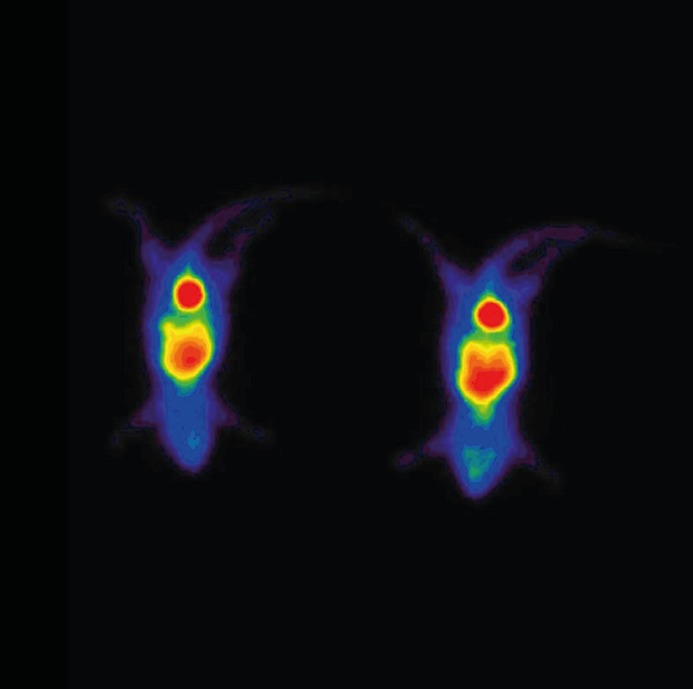
Planar SPECT imaging of the [^99m^TC]-ECB-DG complex after 2 hours administration

Results of the biodistribution studies of these complexes are summarized in tables 2 and 3. [^99m^Tc]-ECB-DG and [^99m^Tc]-DHP-DG complexes showed brain and heart uptake (0.61%±0.09 and 1.16±0.10) at 60 min post- injection respectively, an efficient clearance from the blood, a rapid excretion to the urine and a low retention in the liver and kidneys. Compared to the ^18^F-FDG (25,26), [^99m^Tc]-ECB-DG displayed a 2.8-fold reduction in brain uptake (1.7 ± 0.2 versus 0.61% ± 0.09) whereas ,[^99m^Tc]-DHP-DG just showed 1.9-fold reduction in heart uptake (2.2 ± 0.05 towards 1.16±0.10) at 1 h post-injection. Therefore, these results indicate that ECB-DG and DHP-DG analogues could be used as brain and heart imaging agent respectively. Planar SPECT imaging of the [^99m^Tc]-ECB-DG complex was obtained at 2 h after administration of 4 in normal mouse (Figure 4). On the basis of SPECT image, it seems that this complex have a moderate brain uptake.

**Table 2:** Biodistribution of [^99m^Tc]-ECB-DG in normal mice (n = 3) at 10, 30 and 60 min post-injection^a^

**Tissue**	**[** ^99m^ **Tc]-ECB-DG**			^18^ **F-FDG** ^b^
	10 min	30 min	60 min	60 min
Brain	0.83 ± 0.15	0.69 ± 0.11	0.61 ± 0.09	1.7 ± 0.2
Kidney	5.12 ± 0.22	5.75 ± 0.18	6.30 ± 0.20	7.7 ± 4.0
Lung	4.33 ± 0.18	4.08 ± 0.26	3.83 ± 0.30	2.9 ± 0.6
Heart	1.12 ± 0.21	0.96 ± 0.19	0.80 ± 0.11	2.2 ± 0.05
Spleen	0.63 ± 0.15	1.13 ± 0.27	1.28 ± 0.21	1.4 ± 0.2
Stomach	1.59 ± 0.21	1.69 ± 0.25	1.77 ± 0.34	1.6 ± 0.3
Bowel	5.33 ± 0.30	7.28 ± 0.21	8.50 ± 0.31	2.2 ± 0.4
Liver	14.40 ± 0.41	16.67 ± 0.17	18.10 ± 0.21	3.0 ± 0.3
Blood	2.88 ± 0.18	2.29 ± 0.14	2.10 ± 0.11	---

^a^Expressed as %ID per gram. Each value represents the mean *± *SD. for 3 animals at each interval.

^b^Biodistribution of ^18^F-FDG in different organs of nude mice after 60 min post-injection (n = 12) (25, 26).

**Table 3:** Biodistribution of [^99m^Tc]-DHP-DG in normal mice (n = 3) at 10, 30 and 60 min post-injection^a^

**Tissue**	**[** ^99m^ **Tc]-ECB-DG**			^18^ **F-FDG** ^b^
	10 min	30 min	60 min	60 min
Brain	0.13 ± 0.10	0.11 ± 0.07	0.10 ± 0.02	1.7 ± 0.2
Kidney	4.20 ± 0.32	4.68 ± 0.11	4.92 ± 0.09	7.7 ± 4.0
Lung	1.68 ± 0.15	1.53 ± 0.28	1.38 ± 0.19	2.9 ± 0.6
Heart	1.61 ± 0.22	1.28 ± 0.12	1.16 ± 0.10	2.2 ± 0.05
Spleen	0.47 ± 0.16	0.97 ± 0.25	1.12 ± 0.26	1.4 ± 0.2
Stomach	1.73 ± 0.27	1.83 ± 0.24	1.91 ± 0.21	1.6 ± 0.3
Bowel	11.45 ± 0.33	13.40 ± 0.23	14.62 ± 0.18	2.2 ± 0.4
Liver	18.05 ± 0.12	20.42 ± 0.11	21.85 ± 0.09	3.0 ± 0.3
Blood	1.50 ± 0.12	0.91 ± 0.11	0.73 ± 0.08	---

^a^Expressed as %ID per gram. Each value represents the mean *± *S.D. for 3 animals at each interval.

^b^Biodistribution of ^18^F-FDG in different organs of nude mice after 60 min post-injection (n = 12) (25, 26).

## Conclusion

In summary, two new DG derivatives were synthesized and labeled with technetium-99m-carbonyl successfully. In our studies, [^99m^Tc]-ECB-DG and [^99m^Tc]-DHP-DG were synthesized and their labeling took place at biological pH. Low molecular weight accelerated clearance from blood, and different linkers and chelate cores changed the excretion path. [^99m^Tc]-ECB-DG shows moderate brain uptake (0.61%±0.09) and [^99m^Tc]-DHP-DG shows good heart uptake (1.16±0.10) at 1 h post-injection respectively. On the basis of our results, it seems that ECB-DG and DHP-DG analogues could be used as brain and heart imaging agents respectively. Related work is underway in our laboratory and will be reported in due course.
